# Integrated Method for Purification and Single-Particle Characterization of Lentiviral Vector Systems by Size Exclusion Chromatography and Tunable Resistive Pulse Sensing

**DOI:** 10.1007/s12033-017-0009-8

**Published:** 2017-05-31

**Authors:** Susanne Heider, Julien Muzard, Marianne Zaruba, Christoph Metzner

**Affiliations:** 1 Institute of Virology, University of Veterinary Medicine, Veterinärplatz 1, 1210 Vienna, Austria; 2 Izon Science, 8C Homersham Place, PO Box 39168, Burnside, Christchurch, 8053 New Zealand; 3 Biological Physics, Department of Physics, Chalmers University of Technology, 412 96 Gothenburg, Sweden

**Keywords:** Viral vectors, Virus-like particles, Lentivirus, Extracellular vesicles, Gene therapy, Vaccine development, Size exclusion chromatography, Tunable resistive pulse sensing, Single particle analysis

## Abstract

Elements derived from lentiviral particles such as viral vectors or virus-like particles are commonly used for biotechnological and biomedical applications, for example in mammalian protein expression, gene delivery or therapy, and vaccine development. Preparations of high purity are necessary in most cases, especially for clinical applications. For purification, a wide range of methods are available, from density gradient centrifugation to affinity chromatography. In this study we have employed size exclusion columns specifically designed for the easy purification of extracellular vesicles including exosomes. In addition to viral marker protein and total protein analysis, a well-established single-particle characterization technology, termed tunable resistive pulse sensing, was employed to analyze fractions of highest particle load and purity and characterize the preparations by size and surface charge/electrophoretic mobility. With this study, we propose an integrated platform combining size exclusion chromatography and tunable resistive pulse sensing for monitoring production and purification of viral particles.

**Electronic supplementary material:**

The online version of this article (doi:10.1007/s12033-017-0009-8) contains supplementary material, which is available to authorized users.

## Introduction

Several virus families can be employed for biotechnological or biomedical applications, among them adenoviruses [1, 2], adeno-associated viruses [3], and retro- or lentiviruses [4]. Lentivirus (LV) vectors, derived mostly from human immunodeficiency virus (HIV), are widely used for research (e.g., to express recombinant proteins or generate transgenic animals) [5] and biomedical applications (e.g., in gene therapy or for vaccine development) [6, 7]. Preparations need to be appropriately pure for any envisaged use. Purification strategies for LV particles include density gradient ultracentrifugation, ultrafiltration, precipitation, and different chromatography approaches including affinity-based systems [8–11]. Choice of the method is usually dictated by the stability of the virus, scalability, available infrastructure, and economic considerations.

**Fig. 1:**
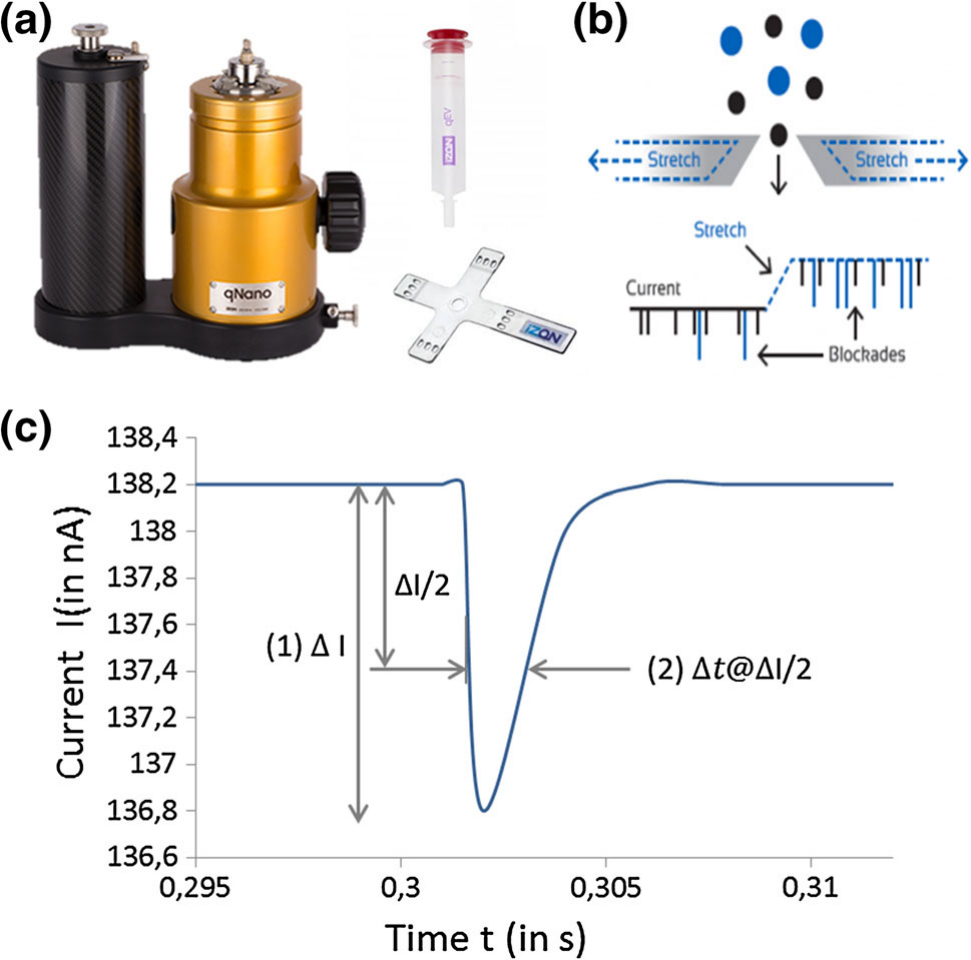
SEC (size exclusion chromatography) qEV column and TRPS instrument (qNANO). **a** The figure depicts the TRPS device (*left*), the qEV column (*right*, *top*) and a cruciform membrane used for TRPS (*right*, *bottom*). The single pore is located in the center of the membrane. **b** The schematic depicts the principle of TRPS. A stretchable pore is traversed by different numbers of particles of different size and charge thus displacing buffer and changing impedance, resulting in resistive pulses also termed blockades. **c** The frequency of resistive pulses provides information about the number of particles, while the height (the magnitude) of the pulse provides information about the volume of the particle. Additionally, the width of the pulse, commonly measured as full width at half maximum magnitude (FWHM) defined as Δ*t* at Δ*I*/2, provides information about the particle speed in the electric field (its electrophoretic mobility) and subsequently its zeta potential

Additionally, quality and quantity of the final preparations (and intermediate products) needs to be monitored [12], ideally in real time and with the possibility to measure multiple parameters (such as size, charge and concentration), independent of the vector (sub-)type. For optimal quality control, single-particle analysis (SPA) would be preferred, since they allow following the distribution of key parameters (e.g., size) in the population, giving information about its heterogeneity [13]. Different approaches to SPA are in use (for comparison of methods see [13, 14] and [15], among them electron microscopy (EM) and dynamic light scattering (DLS) approaches [16], nanoparticle tracking analysis (NTA) [11, 14, 17, 18], flow virometry (FV) [19, 20], and tunable resistive pulse sensing (TRPS) [13, 21, 22]. The latter is based on the Coulter principle, which states that particles pulled through a (physical) pore, while an electric current is applied, produce a change in impedance that is proportional to the volume of the particle traveling through the pore. Coulter-based technologies, known collectively as resistive pulse sensing (RPS), are able to provide a particle-by-particle analysis in situ as individual particles are driven through pores by a combination of electrophoretic, electro-osmotic, and gravitational forces. RPS has been demonstrated to be useful in many fields, including biological detection and particle characterization [23]. In brief, a single pore in a non-conductive membrane separates two electrolyte fluid chambers with an electrode in each; the electrodes establish a stable baseline current, and sample is loaded into one of the fluid chambers. As the sample moves through the pore, deformations in the baseline current occur (“blockade events”) (see Fig. 1). By monitoring blockade event numbers (events/min; indicative of particle concentration), magnitudes (Δ*I*; indicative of the buffer displacement by the particles’ volume) and changes in blockade width Δ*t* (or Δ*t* at Δ*I*/2, the full width at half -maximum FWHM, indicative of the duration of the particles’ pore transfer, i.e., their electrophoretic mobility), it is possible to elucidate the zeta potential, size, and concentration of colloidal dispersions in situ (see Fig. 1b, c). The use of polyurethane and elastomeric membranes in conjunction with RPS has allowed the creation of tunable resistive pulse sensing (TRPS) which by now is an established technique for the characterization of biological and non-biological nanoparticles. In TRPS, the pore can be mechanically manipulated in real time to alter pore geometry and investigate a range of particle sizes with a single pore in addition to allowing significant optimization and the removal of blockages. Different pores are available and allow measuring in a size range from 40 nm to 11.3 µm in the nanopore range and from 5 to 200 µm in the micropore range. Depending on pore size, different target concentrations are recommended, leading to an overall range from 1E05 to 1E11 particles per ml. Images and a schematic of the instrumentation are displayed in Fig. 1. TRPS has been successfully used to study the concentration, size and charge of colloidal dispersions [24] as well as monitoring the concentration-dependent aggregation of superparamagnetic beads [25], the aggregation of nanorods [26], microbubbles [27], and DNA-modified nanoparticles [28]. A newer field of application for TRPS is the characterization of biological submicron lipid enclosed vesicles. To date, extracellular vesicles derived from eukaryotic cells (e.g., exosomes) [29–32], bacteria [33], and archaea [34] have been analyzed by TRPS, providing reliable size and concentration data as well as viral particles from diverse species such as vesicular stomatitis virus [35], the arenavirus Junin [37], a rotavirus [37], and HIV-like particles [7]. Indeed, similar to exosomes in size (approximately 100 nm), LV particles seem a logical step in the analysis of bio-vesicles by RPS due to their widespread use as gene therapy vectors.

As a model system LV producer cell lines based on the STAR system generating HIV-like particles optionally modified with a retroviral surface glycoprotein from murine leukemia virus (amphotropic 4070A MLV Env) [38, 39] were used, to see whether differences can be determined as a result of the presence of the viral surface protein. To our knowledge, LV particles (with and without Env) were subjected to the size exclusion chromatography (SEC) purification system developed for extracellular vesicles, termed qEV, for the first time. Using TRPS we have shown that the size, concentration, and electrophoretic mobility of the viral preparations can be measured in a quick and straightforward fashion. We have successfully extended the range of analytes to LV and herpes virus particles. In this report, we thus demonstrate the potential of a single workflow, combining SEC by qEV and TRPS in monitoring the production of LV (e.g., for size, concentration).

## Materials and Methods

### Viral Vector Preparation

STAR and STAR-A-HV (ECACC Nos. 04072119 and 04072115, respectively) are lentiviral producer cell lines based on HEK293T cells. Herpesvirus preparations were produced by de novo infection of CrFK cells (ATCC accession CCL-94™) with feline herpesvirus type 1 (FHV-1), as described previously [40]. Cells were cultured in DMEM medium supplemented with 10% fetal bovine serum. Concentration of viruses previous to purification was either carried out by ultracentrifugation or by ultrafiltration as described previously [40].

### Size exclusion Purification of Vector Preparations

For purification of virus samples, commercially available size exclusion columns (qEV, Izon Science, New Zealand) were used. The procedure was carried out as described by the manufacturer. In brief: 0.5–1 ml of clarified cell culture medium (centrifuged at 2200×*g* and filtered through 0.45-µm syringe filters) was overlaid on qEV size exclusion columns (Izon Science Ltd, New Zealand) followed by elution with PBS. 0.5 ml fractions were collected (and used immediately or stored at −80 °C before further analysis). The columns contain 10-ml resin material (with a pore size of approximately 75 nm) and has a void volume of 3 ml and a nominal separation size of 70 nm.

### Total Protein Concentration Measurement

Levels of protein were determined using the BioRad ProteinDC quantification kit according to the manufacturer’s instructions. Samples were analyzed in microtiter plates using a Tecan Genios plate reader.

### Silver Staining

Samples were subjected to SDS-PAGE using 10% gels and a Laemmli buffer system. Silver staining was carried out as described previously [41].

### p24 Immunoblotting

Samples were subjected to SDS-PAGE using 10% gels and a Laemmli buffer system. Proteins were electro-transferred onto PVDF membranes (GE Healthcare) and incubated o/n in blocking buffer (4% milk powder w/v; 1% bovine serum albumin w/v in tris-buffered saline containing 0.1% Tween 20). Primary antibody purchased from Polymun (Vienna, Austria) was used in a 1:1000 dilution. HRP-labeled anti-murine secondary antibody (from DakoCytomation) was used in dilutions of 1:10,000. ECL detection kits (GE Healthcare) were used for generating signals, which were developed and recorded using an AGFA Curix 60 developer and film (GE Healthcare).

### Product-Enhanced Reverse Transcriptase (PERT) Assay

PERT assays infer viral particle numbers from the levels of reverse transcriptase enzyme activity. Assays were carried out as described previously [40].

### Tunable Resistive Pulse Sensing (TRPS) Analysis

All measurements were conducted using the qNANO (Izon Science Ltd., New Zealand). The lower fluid cell always contained the electrolyte buffer (75 μl). The upper fluid cell always contained 40 μl of sample (that was suspended in the buffer) when a measurement was being completed with pressure. Prior to TRPS analysis, all samples were vortexed for 30 s. Between each sample run, the system was washed by placing Izon Science’s Solution Q or PBS containing 0.1% Tween 20 (40 μl) into the upper fluid cell several times with various pressures applied to ensure there were no residual particles remaining and therefore no cross contamination between samples. To ensure the most efficient measurements, all solutions are filtered using 0.45-µm filters before use. Also, degassing of all solutions is recommended. Increased purity will facilitate measurements. A detailed description of such a tunable resistive pulse sensing device can be found in Willmott et al. and Vogel et al. [21, 25, 29, 42]. The concentration, size distribution, and electrophoretic mobility of particles were analyzed using a NP100 nanopore (Izon Science Ltd., New Zealand) at a 45-mm stretch. The choice of the right nanopore size is critical for efficient measurements. The concentration of particles was standardized using multi-pressure calibration with 70-nm carboxylated polystyrene beads at a concentration of 1.5 × 10^11^ particles/ml (Izon Science Ltd., New Zealand).

### Calculations and Software

Relative data were calculated by dividing absolute values by the levels in the starting material and multiplying by one hundred to obtain percentages. Recovery rates were determined by combining the values from fractions containing the most viral markers and dividing by levels in the starting material, followed by multiplication with one hundred to obtain percentages. Purity was calculated by dividing a measure of concentration of viral marker with a measure of total protein content, providing a measure for viral marker per µg total protein. The higher the value, the more pure is the preparation. Enrichment is calculated by dividing the purity value of a given fraction by the purity value of the starting material. All TRPS measurements were processed with proprietary data capture and analysis software (Izon Control Suite v.3.3). Densitometry analysis on p24 immunoblots and silver-staining images were carried out using ImageJ [43].

## Results

### Biochemical Analysis of Purified Virus

**Fig. 2:**
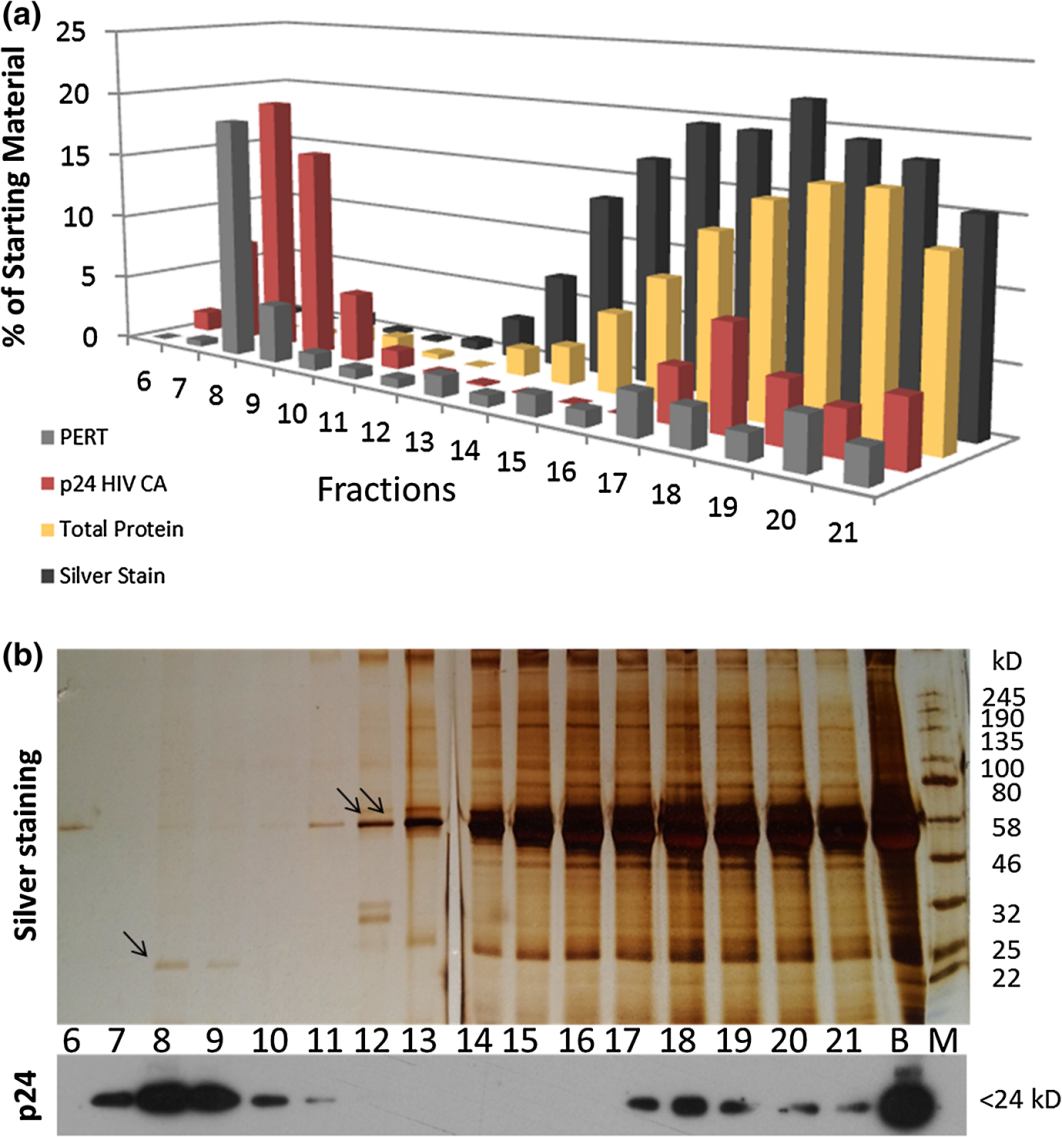
Biochemical characterization of qEV purified lentiviral preparations. **a** Aliquots taken from the starting material before qEV treatment (*B*) and from the fractions during the procedure (*6–21*) were analyzed by PERT assay (*gray columns*), immunoblot for p24 HIV CA protein (*red columns*), a total protein detection method based on a modified version of Lowry’s assay (*yellow columns*) and silver staining (*black columns*). Data depicted are relative to starting material (in %). While virus-specific markers (PERT and p24) are most prominent in early fractions (*7–10*) total protein is detected mainly later, clustered around fraction *18*. **b** The *top panel* shows a representative silver-stained PAGE gel, again signals indicating the presence of different protein species are significantly denser in the higher fractions clustering around fraction *18*. *Single arrow* indicates a signal at approximately 24 kD most likely corresponding to the specific signal detected for the most abundant viral protein HIV p24 CA. The *double arrow* indicates the most abundant contamination found at approximately 60 kD. This signal most likely corresponds to albumins. The *lower panel* shows results from an immunoblot staining specific for the HIV p24 CA protein. Signals are found in earlier fractions (*7–10*) *underlining* the particulate nature of the sample

In a first round of experiments, we analyzed samples by biochemical methods, to determine distribution of total protein and virus associated proteins over the fractions collected in the qEV procedure (see Fig. 2a). Total protein levels were determined by a modified version of the Lowry method (see Fig. 2a, yellow columns) and by silver staining (see Fig. 2a, black columns) to get an overview of the presence of different protein species. Additionally levels for two viral proteins were determined: the lentiviral capsid (CA) protein derived from the *gag* gene (p24) (see Fig. 2a, red columns) and the archetypical retroviral enzyme reverse transcriptase (RT) derived from *pol* which enables the conversion of RNA to DNA during the viral replicative cycle (see Fig. 2a, gray columns). When looking at the distribution of total protein amounts over the collected fractions, the data from both methods (Silver and modified Lowry) showed that the vast majority of proteins was present in the later fractions (see Fig. 2a, black and yellow columns, respectively) while both virus-specific markers (p24 and RT) were predominantly found in the early fractions (see Fig. 2a, red and gray columns). The data are represented as values relative to levels before qEV purification. Absolute data can be found in supplementary information (see Supplementary Fig. 1). For p24 and silver staining data, representative pictures are shown. Again, the majority of virus-related markers are found in early fractions (mostly 7–10) while total protein is concentrated in fractions clustered around fraction 18 (see Fig. 2b). Albumin, around the molecular weight band of 58 kD (see Fig. 2b, indicated by double arrows), and a signal at roughly 25 kD most likely corresponding to p24 are clearly visible (see Fig. 2b, indicated by a single arrow).

### Concentration, Size Distribution, and Charge Analysis of Virus by TRPS

**Fig. 3:**
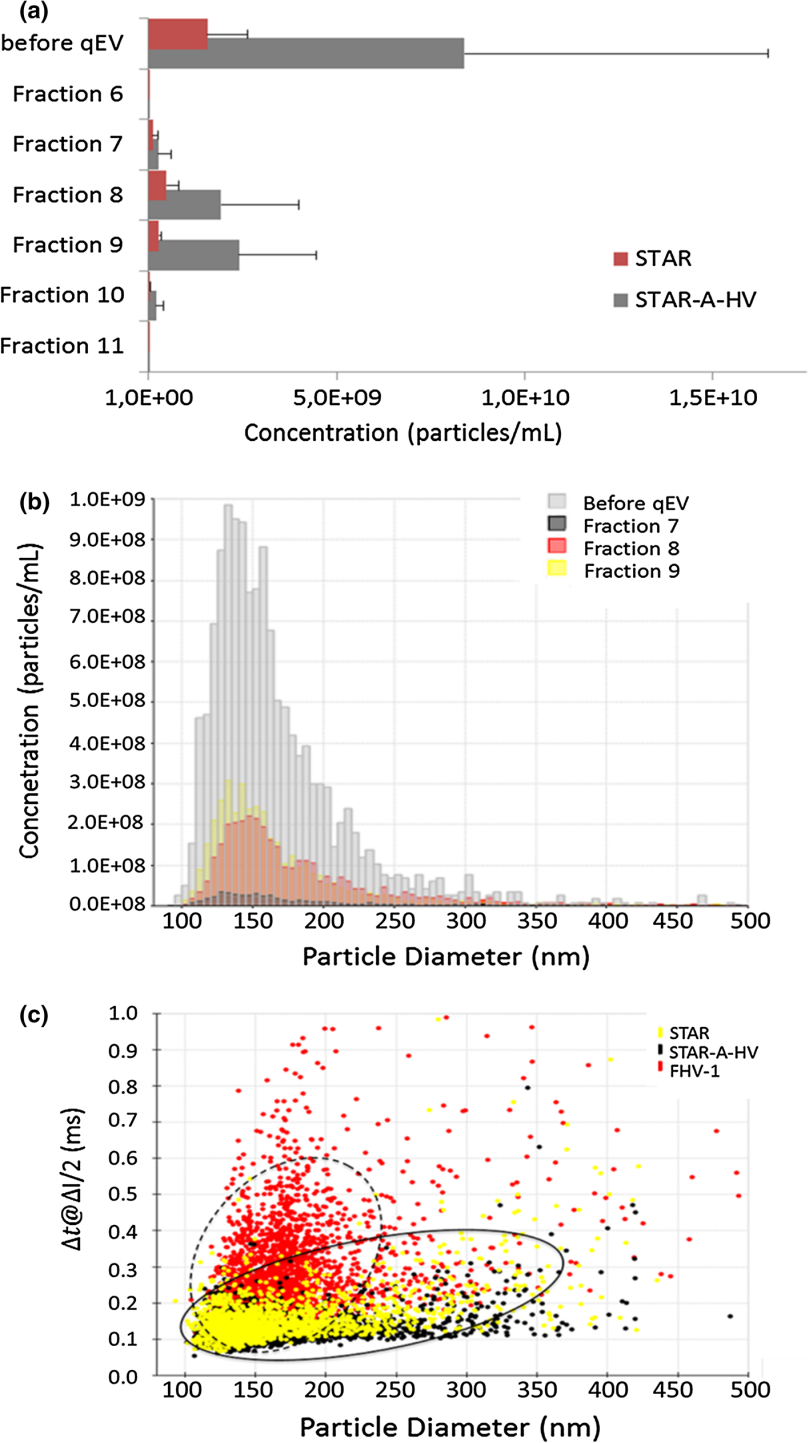
TRPS concentration/size/charge. **a** The graph depicts concentration measurements for selected fractions from qEV purifications. While samples produced from the STAR cell line (*red bars*) usually gives lower titers than those produced from STAR-A-HV cells (*gray bars*), the distribution remains the same. The highest concentrations of particles are found in *fractions 8* and *9*. *Bars* show average and standard deviations of two independent experiments. **b** The graph depicts size distribution measurements from different fractions of a STAR-A-HV viral preparation. Other than the difference in the number of counts, no significant changes in the distribution are observed. **c** The graph depicts charge analysis carried out on the qNANO. Three different virus populations were analyzed: STAR and STAR-A-HV derived particles (*yellow* and *black dots*, respectively) as well as FHV-1 particles (*red dots*). While no difference can be seen between the lentiviral preparations from STAR and STAR-A-HV cells (see *full ellipsoid*), the herpesviral FHV-1 virus shows a different pattern, shifted to longer blockade durations (see *broken ellipsoid*)

In addition to the biochemical data collected, we analyzed selected fractions for particle content. In addition to particle number, TRPS also allows for the measurement of viral titer, diameter and zeta potential (see Figs. 1, 3). We were predominantly interested in the fractions which contained the highest levels of virus according to biochemical analysis (fractions 7–10). As control, also the samples before qEV (see Fig. 3a, b, samples Before qEV) were tested. We analyzed lentiviral particle preparations derived from STAR and STAR-A-HV producing cells for all of these parameters. The distribution of particle concentration was comparable to data obtained from the biochemical analysis. While in fractions 7, 11, and 12 very few particles were found, most particles were found in fractions 8–10 (see Fig. 3a). Results for STAR and STAR-A-HV particles showed little difference (compare Fig. 3a, columns STAR and STAR-A-HV). In terms of particle size and distribution, we observed very little difference between the two viral variants and good reproducibility (see Table 1; Fig. 3b, respectively). When charge analysis was carried out according to the specifications described in the Materials and methods section, again, no significant difference was visible between STAR and STAR-A-HV derived viral particles (see Fig. 3c). As charge control we added a herpes viral preparation that was treated with the reducing agent dithiothreitol (DTT), and indeed a difference in zeta potential could be observed (see Fig. 3c, compare yellow, black and red dots).

**Table 1:** Size characteristics

Virus	Mean (nm)	Mode (nm)
STAR	178 ± 23	145 ± 13
STAR-A-HV	178 ± 7	137 ± 7
FHV-1	223 ± 1	222 ± 1

Date are represented as averages ± SD

### Recovery and Purity of qEV-Purified Lentiviral Samples

To obtain an estimate of the efficiency of the method we determined measures of purity and recovery rates (see Tables 2, 3). Recovery was defined as the cumulative marker amount in the key fractions relative to the total starting amount (see also “Materials and Methods”). Depending on the technique used for measuring the parameter, values ranged from 24.47% (by PERT) and 48.13% (by p24) to 57% (by TRPS) (see Table 2). The observed differences reflect the different properties of the measured parameters (for more information see discussion section). Purity was defined as the quantitative measure of a viral marker (PERT or p24) relative to the amount of total protein content as measured by modified Lowry assay: the more viral marker and the less total protein are present in the fraction, the greater is the purity. The enrichment is describing purity relative to the starting material. For the strongest fraction 8, enrichment was in good agreement with both methods (60.35- and 57-fold increase in purity over the starting material, respectively, see Table 3). Again, differences and irregularities are due to the measured entities and the used techniques and will be discussed later.

**Table 2:** Recovery

Recovery by	in %
PERT	24.47
p24	48.13
TRPS	57.18

**Table 3:** Purity of selected fractions

Fraction	By p24/total protein^a^	Enrichment^b^	By PERT/total protein^c^	Enrichment2^b^
B	0.12	1.00	192.10	1.00
7	31.61	268.21	3069.83	15.98
8	7.11	**60.35**	10950.62	**57.00**
9	1.65	14.01	752.73	3.92
10	0.56	4.72	218.95	1.14

^a^Relative signal intensity per ng total protein

^b^Compared to B

^c^Particles per ng total protein

## Discussion

Our studies using qEV columns indicated the potential for this method in the fractionation (and subsequent characterization) of lentiviral preparations by size exclusion. A clear separation effect between viral proteins and bulk total proteins was observed, probably most vividly recognizable in images from silver-stained PAGE gels (see Fig. 2b, upper panel). Signals for virus-specific markers from PERT and immunoblots are also observed in the higher fraction, indicating lower molecular weight components. These represent viral debris or non-aggregated proteins, not associated with or organized in complete virion particles [37]. Quantitative differences between these two peaks (particulate and debris) can be indicative of the quality of a viral preparation. The more particulate and the less debris viral markers are observed, the higher the quality of the preparation is.

TRPS data for viral concentration matches well with the biochemical analysis, i.e., fractions 8 and 9 contain the greatest number of particles (see Fig. 3a). The higher numbers observed for the STAR-A-HV derived particles compared to STAR particles are also reflected in the starting material (see Fig. 3a, columns before qEV). This may either be a consequence of the production capabilities of the respective producing cell lines, or reflect stability of the particles. The lack of the MLV 4070A Env protein may weaken the integrity of the viral envelope. In addition to this, while the size of particles derived from STAR and STAR-A-HV cells is quite similar, the observed standard deviation is increased for STAR compared to STAR-A-HV derived particles (see Table 1). Again, this may suggest greater heterogeneity and/or decreased stability of the Env-less viral variant. When compared to a different method for single particle analysis, NTA, both results and observed challenges were comparable. Representative outcomes for two viral preparations (STAR and FHV-1) are depicted in Supplementary Fig. 2. Size measurements agree exceptionally well: for STAR a mode diameter of 145 nm by TRPS is matched by 131 nm by NTA; for FHV-1 the respective values are 222 nm (TRPS) and 216 nm (NTA) (see also Table 1 and Supplementary Fig. 2). For further comparison of SPA techniques, additional recent information is available [13, 15].

An interesting side aspect emerged from the analysis of electrophoretic motility. The charge, or zeta potential (as measured by DLS and RPS) of enveloped viruses, such as the lenti- or herpesviruses used in the study is considered low (roughly at ±5 mV) [44]. Alternatively, isoelectric points can be determined for intact virus particles [45]. Not surprisingly, there was no significant difference observed between STAR and STAR-A-HV derived particles because they only differ in one determinant present in low copy number (the MLV 4070A env; see Fig. 3c, compare yellow and black dots). However, a clear difference was visible between STAR-derived and DTT-treated herpesvirus particles. The blockade duration is shifted toward longer intervals, most likely indicating a reduced electrophoretic mobility as a consequence of reduced zeta potential or charge (see Fig. 3c; compare full and broken ellipsoids, indicating virus populations). This strongly suggests that biochemical differences between viral species and/or chemical changes to viral surfaces can be detected by TRPS. This fact can be further developed as an analytical tool to differentiate between different virus types or states.

Measurement differences especially between TRPS, PERT, and p24 analysis are best seen in recovery rates (see Table 2) and probably indicate the stringency levels of the techniques [13]. While PERT requires a functional RT molecule, presence of the p24 capsid protein epitope is sufficient for detection. In TRPS measurements, all particles of a certain size are counted, irrespective of their origin. The good agreement of data between TRPS and p24 recovery rates (57–48%) indicates that the vast majority of counted particles are indeed viral particles. Enrichment values for the fraction eight containing most viruses were in good agreement (60–57%) for the two measurement methods (see Table 3, in bold). However, other fractions do not agree (i.e., fractions 7 or 9). When measured values get smaller, the quantitative content of the measurement is reduced. This is especially true for the p24 immunoblot density data. This introduces a stronger variation in the weaker bands, most likely leading to the irregularities in enrichment data.

In this study we have demonstrated the application of commercially available SEC columns (Izon science qEV) to the purification of lentiviral preparations. Protein contaminants can be efficiently removed from the samples in a fast and cost-friendly manner. Additionally, we employed TRPS (Izon qNANO) to characterize viral preparations for titer, size and electrophoretic mobility simultaneously, demonstrating important potential for virus production processes.

## Electronic supplementary material

Below is the link to the electronic supplementary material.

Supplementary material 1 (PDF 494 kb)

## Abbreviations

FV: Flow virometry
LV: Lentivirus
NTA: Nanoparticle tracking analysis
PERT: Product-enhanced reverse transcriptase assay
RPS: Resistive pulse sensing
SEC: Size exclusion chromatography
SPA: Single-particle analysis
TRPS: Tunable resistive pulse sensing
