# Supplementary material for: Integrated Method for Purification and Single-Particle Characterization of Lentiviral Vector Systems by Size Exclusion Chromatography and Tunable Resistive Pulse Sensing
Source: Mol Biotechnol. 2017 May 31;59(7):251–9. doi: 10.1007/s12033-017-0009-8 (PMC5486506; doi:10.1007/s12033-017-0009-8)
Supplement: Supplementary file 1 — Supplementary material 1 (PDF 494 kb) [file 12033_2017_9_MOESM1_ESM.pdf]

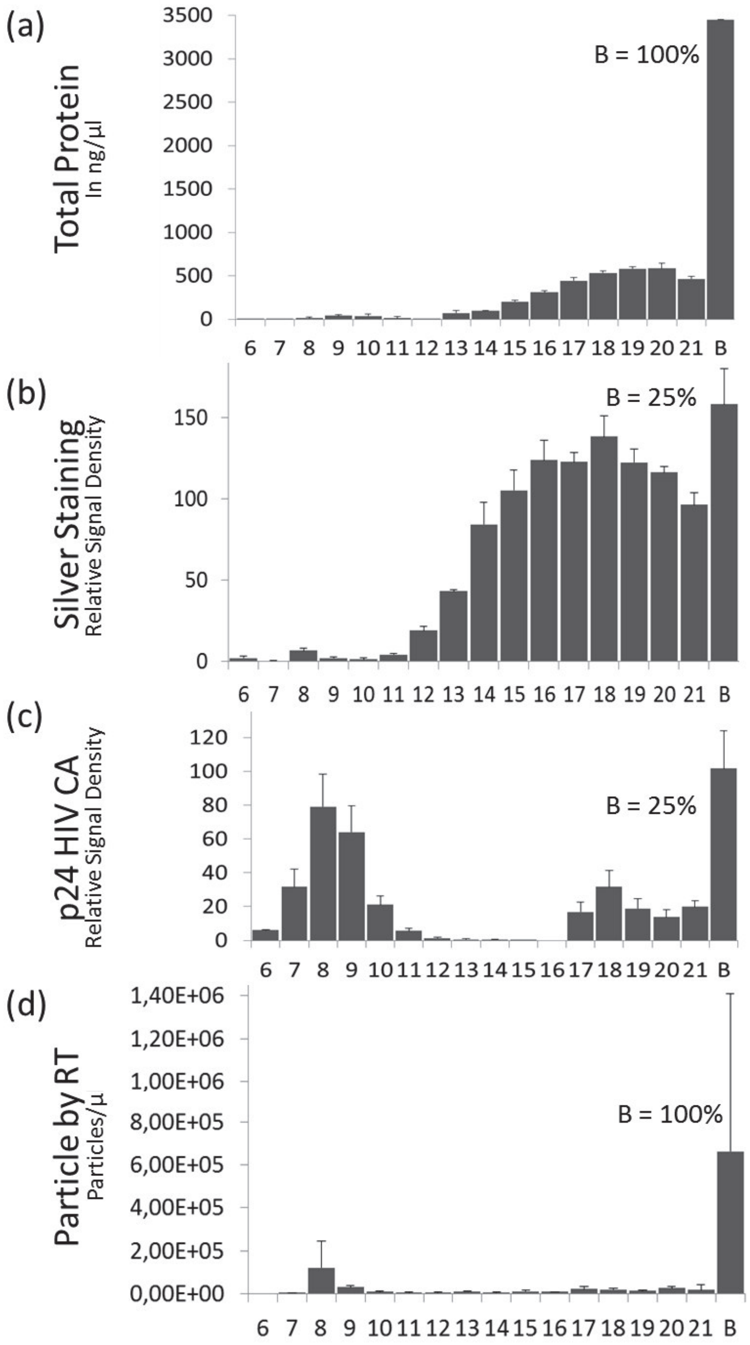

Supplementary figure 1. Biochemical analysis raw data. The graphs depict raw data derived from (a) total protein, (b) silver staining and (c) p24 densitometry analysis as well as (d) PERT measurements. Data is represented as averages (n≥2). Error bar indicates standard deviation. While total protein is dominant in late fractions (starting at 12), virus specific protein is dominant in earlier fractions

(mainly 8 and 9). The percentages indicated for the before qEV (B) samples denote the fraction of starting material used in the assay.

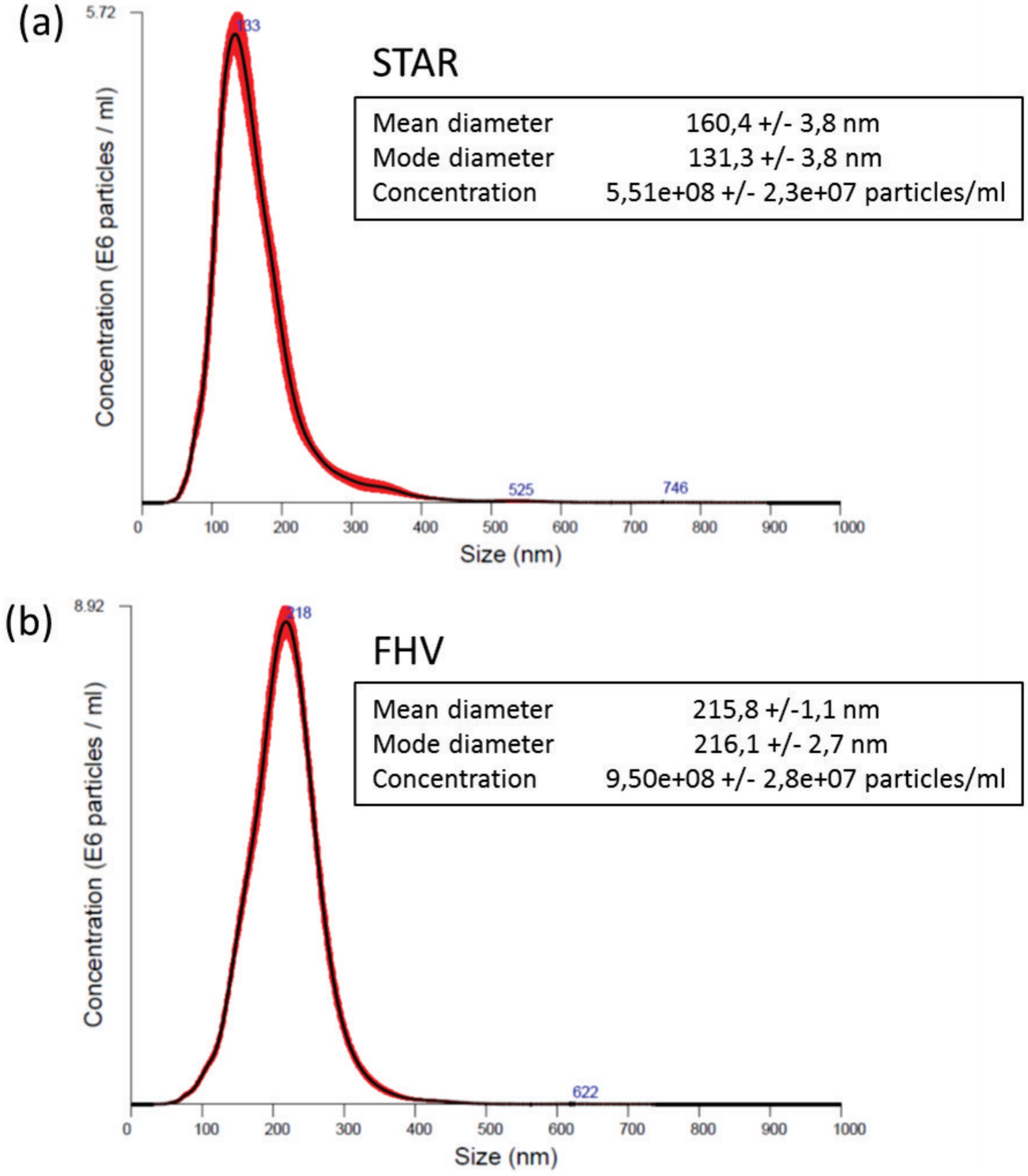

Supplementary Figure 2: Nanoparticle tracking analyis of (a) STAR and (b) FHV-1 virus preparations. 5 measurements were averaged for the figure. All measurements were conducted on a Nanosight NS500 device and analyzed using NTA 3.0 0068 version. Sample preparation was conducted as described in the material and methods section. Size distribution, mean and mode of diameter are shown as well as the concentration of the preparation. Red area indicates the +/-1 % standard error of the mean. Blue numbers indicate local maxima of size distribution.
